# Supplementary material for: Disrupted Pallido-Thalamo-Cortical Functional Connectivity in Chronic Disorders of Consciousness
Source: Brain Sci. 2021 Mar 11;11(3):356. doi: 10.3390/brainsci11030356 (PMC7999530; doi:10.3390/brainsci11030356)

brain sciences

MDPI

Supplementary Materials:

Table S1. Functional connectivity statistics.

**Controls**		**T-score**	**p-FDR corrected**
**Thalamus R**	Thalamus L	14.56	0.000000
	SN – supramarginal gyrus R	6.24	0.000288
	Pallidum R	5.39	0.001109
	CEN – posterior parietal cortex R	4.85	0.001950
	SN – anterior insula R	4.06	0.009382
	SN – anterior cingulate cortex	3.86	0.012766
	SN – supramarginal gyrus L	3.59	0.016455
	SN – rostral prefrontal cortex R	3.06	0.043071
	SN – anterior insula L	2.93	0.046804
**Thalamus L**	Thalamus R	14.56	0.000000
	Pallidum L	5.29	0.001191
	CEN – lateral prefrontal cortex L	4.36	0.005456
	SN – anterior insula L	4.20	0.006385
	SN – anterior cingulate cortex	4.01	0.008170
	CEN – lateral prefrontal cortex R	-3.61	0.015674
	SN - supramarginal gyrus L	3.56	0.016949
	Language network – inferior frontal gyrus L	3.34	0.025062
	Visual network – medial	-3.14	0.030241
	CEN – posterior parietal cortex R	-2.98	0.041191
	Pallidum R	2.94	0.044330
	SN – rostral prefrontal cortex L	2.91	0.046225
	Sensorimotor network – lateral R	-2.87	0.049902
**Pallidum R**	Pallidum L	12.34	0.000000
	SN – anterior insula R	9.22	0.000002
	SN – anterior insula L	5.68	0.000400
	Thalamus R	5.39	0.000665
	SN – anterior cingulate cortex	4.45	0.004080
	SN – supramarginal gyrus R	4.19	0.006118
	SN – rostral prefrontal cortex R	3.68	0.015783
	DMN – lateral parietal cortex L	-3.31	0.033483
	Thalamus L	2.98	0.049056
	SN – supramarginal gyrus L	2.96	0.049737
**Pallidum L**	Pallidum R	12.34	0.000000
	SN – anterior insula L	7.78	0.000015
	Thalamus L	5.29	0.001389
	SN – anterior cingulate cortex	4.36	0.007766
	SN – anterior insula R	4.13	0.009390
	SN – rostral prefrontal cortex L	3.49	0.024231
	Cerebellar network – anterior	3.36	0.028916
	DMN – lateral parietal cortex R	-3.30	0.029830
	DMN – medial prefrontal cortex	-3.26	0.029830
	DMN – lateral parietal cortex L	-3.20	0.032870
	DMN – precuneus/retrosplenial cortex	-2.99	0.045047
	SN – rostral prefrontal cortex R	2.97	0.045361

**Patients**		**T-score**	**p-FDR corrected**
**Thalamus R**	**Thalamus L**	5.76	0.005992
**Pallidum R**	Pallidum L	4.62	0.013933
	Language network – posterior superior temporal gyrus R	4.20	0.025645
**Pallidum L**	Pallidum R	4.62	0.012138
	SN – rostral prefrontal cortex R	-3.87	0.040747

**Controls > Patients**		**T-score**	**p-FDR corrected**
**Thalamus R**	SN – anterior cingulate cortex	4.06	0.014447
	SN – supramarginal gyrus R	4.03	0.014447
	SN – rostral prefrontal cortex R	3.88	0.017503
	CEN – posterior parietal cortex R	3.63	0.024537
	SN – anterior insula R	3.33	0.047876
**Thalamus L**	CEN – lateral prefrontal cortex L	4.73	0.002049
	SN – anterior cingulate cortex	4.25	0.005162
	SN – rostral prefrontal cortex L	3.24	0.037225
**Pallidum R**	DMN – lateral parietal cortex R	-3.93	0.038007
	SN – anterior insula R	3.73	0.038694
**Pallidum L**	SN – rostral prefrontal cortex R	4.74	0.007999
	DMN – precuneus/retrosplenial cortex	-4.25	0.015811
	DMN – lateral parietal cortex R	-3.80	0.045887

R, right; L, left; SN, salience network; CEN, executive network; DMN, default mode network.

Figure S1. Grey matter volumes of the cerebral networks, pallidums and thalami in patients (P) and controls (C). The volumes were measured with MRIcron (http://www.nitrc.org/projetcs/mricron), by using the intersection logical tool between, on the one hand, the grey matter masks generated by SPM12's automatic segmentation and, on the other hand, the regions of interests as defined in paragraph 2.3. Volumes were systematically significantly smaller in patients compared to controls, except for the pallidums. Boxplots are made of the first and third quartiles. The minimum (purple), median (bold black) and maximum (red) are indicated. CEN, executive network; DMN, default mode network; SN, salience network; * $p<0.05$ (Mann-Whitney U test).

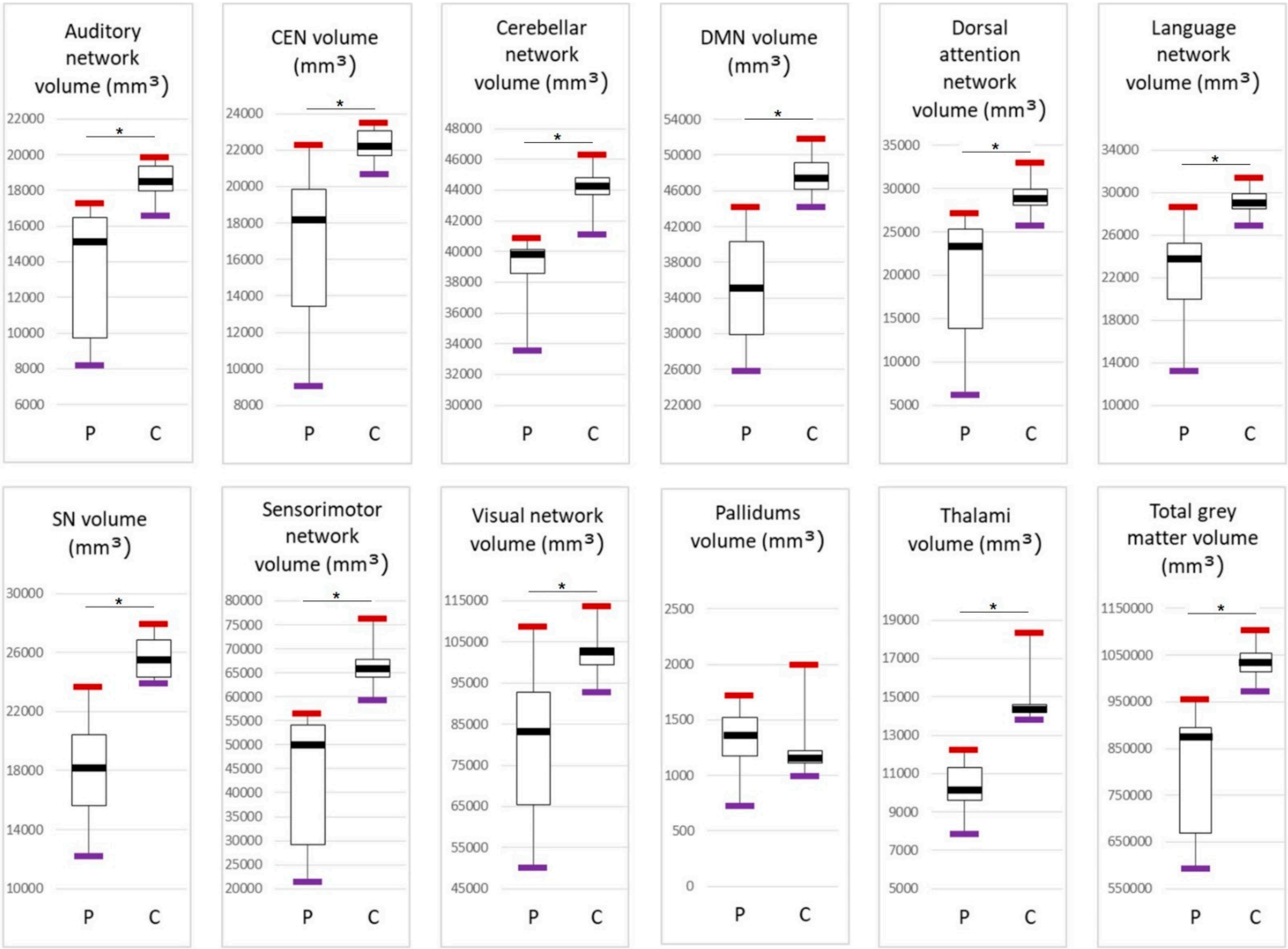

Supplement: Supplementary file 1 [file brainsci-11-00356-s001.pdf]
